# The Relation of Gonorrhea to Pelvic Diseases in Women1In preparing this article, I have drawn largely from the writings of Tait, Sinclair, Longyear, and others.

**Published:** 1893-03

**Authors:** Matthew D. Mann

**Affiliations:** Professor of Obstetrics and Gynecology in the Medical Department of the University of Buffalo


### THE RELATION OF GONORRHEA TO PELVIC DISEASES IN WOMEN.[1]

By MATTHEW D. MANN, A. M., M. D.
Professor of Obstetrics and Gynecology in the Medical Department of the University of Buffalo.

It may seem to some of you that sufficient has already been said on this subject of late years, and that the matter might now be very properly dropped. I know that the journals have been full of articles, and that many of our societies have discussed the matter very thoroughly. But, nevertheless, in my intercourse with the profession I find that a great deal of misapprehension still exists, that many of the most important facts are only imperfectly understood, and that, as a result, a great deal of harm is being done by those who should know how to do better.

1. In preparing this article, I have drawn largely from the writings of Tait, Sinclair, Longyear, and others.

It is exceedingly important that the general practitioners should have this matter made very plain to them, because, as a rule, cases of this disease apply to them for the early treatments, and it is only after they have become bad, or almost incurable, that they apply to the specialist. The general practitioner often has the golden opportunity of eradicating the disease at its start, an opportunity which the specialist very rarely has presented to him. It is for this reason more than for any other that it seems to me that this discussion may be profitable and appropriate.

The importance of the subject cannot possibly be overestimated. The amount of mischief which is done by gonorrhea is almost beyond belief. It is only recently that we have come to appreciate this. But now, I think that we can truly say, in the light of modern discoveries and of an enlarged experience, that gonorrhea does vastly more harm than syphilis. It must be remembered that syphilis is a disease largely amenable to treatment, and that if seen early it can generally be cured, or, at least, its violence greatly mitigated. But in the case of gonorrhea in women, if the disease has once reached the Fallopian tubes or beyond, it is practically beyond our reach except by surgical measures.

Again, gonorrhea is infinitely more common than syphilis. I think I can truly say that for every case of syphilitic disease that I see in my consulting room, there are forty-nine cases of gonorrhea. In this connection let me quote a passage from Mr. Tait:

> Early in life I heard an eminent surgeon, one of my own teachers, say that if he were condemned to have a venereal disease, he would rather have syphilis than gonorrhea. I marveled and disbelieved, but now I know that if he included women in his thoughts of the subject, he spoke truly. Syphilis is, relatively, a harmless disease, may cause discomfort and distress, and even much pain, but I doubt if it ever kills women. If it does, where syphilis kills its tens, gonorrhea kills its thousands, and it would take the sufferings of a hundred cases of syphilis to make up for the long, weary years of agony of one case of gonorrheal pyosalpinx."

Noeggerath and Tait have thrown a flood of light on this subject, and for this the whole profession owes them a debt of gratitude.

But how has the importance of this subject come to be appreciated? In this way: The knowledge of the true pathology of pelvic inflammations has been the stepping stone to it. Formerly, all pelvic inflammations were thought to be cellulitis, but recent researches have shown that the teachings of Goupil and Bernutz were correct, and that the inflammatory troubles around the uterus are not in the cellular tissue, but are, in reality, in the tubes, ovaries, and surrounding peritoneum. Once this fact was admitted, the question arose as to what was the cause of the inflammation of the tubes, and this was soon answered by Noeggerath and Tait, showing that the gonorrheal poison was really at the bottom of it all.

When we look at the anatomy of the uterus and tubes, we can readily understand how very easily the poison can pass from the one to the other. Here we have a distinct line of communication between the vagina on the one side and the abdominal cavity on the other. What wonder, then, that a germ which has the power of spreading itself, as we know very well to be the case in the male, to distant organs by contiguity of surface, should, in the female, follow the line indicated and spread from the vagina, where the poison is first deposited, through the tubes to the peritoneum? At first this was largely conjecture, supported only by clinical evidence, but with the discovery of the specific bacterium, the gonococcus of Neisser, the whole matter became quite clear.

If, then, as is indicated, the majority of pelvic inflammations have their seat in the tubes and ovaries, and if these inflammations are caused by the gonorrheal poison, every one who has practised gynecology cannot fail to appreciate the great importance of this subject.

Now, let us study the course of this disease more in detail. We admit, at the start, that it is a specific inflammation. What tissues can be infected? As in the male, so in the female, the urethra is often the primary seat of the disease. From here it may extend into the bladder, from thence into the ureters, and even to the kidneys. This complication, fortunately, is rare, but I have seen several cases of ureteritis which could only be accounted for in this way.

Besides spreading upward from the urethra, the poison spreads downward to the vulva, or it may have its original seat there, and much of the primary inflammation is often a pure vulvitis. In every case, I think we can say without exception, the vulvo-vaginal glands become infected, and the disease lingers in this locality longer than almost anywhere else.

From the vulva it is generally supposed that the disease spreads upwards into the vagina; but this is, undoubtedly, misapprehension, as vaginitis is certainly one of the rare phenomena of gonorrhea. I wish to impress you fully with this fact, because it is not yet fully understood, and many a woman has been pronounced "clean" in whom the disease still lingered, although the vagina was apparently perfectly healthy.

It is not the vagina, but the cervix, in which this disease manifests its presence most actively, and here it is that the disease has its seat, and here it clings most tenaciously. The reason why the cervix, and not the vagina, is attacked, can be readily understood when we consider the nature of the mucous membrane of the vagina. It is thick, covered with numerous layers of strong epithelium, and offers great resistance to the entrance of the germ. Moreover, the secretion of the vagina is acid, which is not favorable to the growth of the germ. In the cervix, however, the conditions all favor its multiplication; the thin epithelium, an alkaline secretion, and the absence of any interference by injections or anything of that kind. But once having gained its seat in the cervix, there is nothing to hinder it from making its way upward; the conditions within the uterus are equally favoring, and the same is true of the tubes. The spread, therefore, from the cervix through the uterus to the tubes is only a natural sequence. Once in the tubes there is no obstruction to its passage into the peritoneal cavity, but when it reaches the serous membrane there seems to be a decided obstacle to its further progress. Evidently the germ does not grow well on the serous membranes; and once it has reached this location, from the well-known peculiarity of the serous membrane, its seat is soon enclosed by a limiting false membrane, and thus all further progress is hindered. In all these cases, therefore, if the poison has made its way through the tube we find adhesions shutting off and limiting the disease.

So far I have described what may be known as an acute gonorrhea, but there is, unquestionably, another form of the disease, spoken of as the "latent" or "creeping" gonorrhea; and this is the more deadly, because the more insidious. It does not attack the vagina at all, rarely shows itself even in the vulva, but expends its whole force upon the uterus and tubes, so that the patient, without any evidence of ever having had the disease of which she is cognizant, finds herself gradually becoming an invalid from a pelvic inflammation, the nature of which she little suspects. This creeping form is the kind that is propagated from men who have a gleet, or in whom the disease has so far been cured that they are unconscious of any evidences of its existence.

This subject, however, belongs to another to elucidate. I do not propose to give a complete history of gonorrhea, but there are still a few questions which I would like to discuss.

Supposing that the disease has attacked the tubes and ovaries, spreading either from an acute or from a latent form, what are the results? Is it necessarily followed by a production of pus ? From clinical observation I feel very confident that it is not. Many cases of gonorrheal salpingitis recover entirely, nor is it necessary even that the woman should be forever sterile. I had one case under observation in which a young married woman was affected with a severe endocervicitis, soon followed by disease of the tubes and ovaries, which never caused her any great amount of inconvenience, which soon subsided, left no traces of its previous existence, and must have entirely disappeared, for after a time she bore a healthy, living child. Now, this, I admit, is the exception, but it is well to bear in mind its possibility.

In many of the cases we find that the tubes do not contain pus, but rather a fluid, either serum or blood. In some of them the walls are enormously hypertrophied, being in some instances half an inch thick, and in other cases, especially where the tubes contain water, the walls may be very thin.

Should the tube contain pus, the pus is by no means always confined to its original seat. It may gradually leak out, causing with each leakage an acute attack of inflammation, so-called "recurrent pelvic peritonitis," and it may finally make its way into the peritoneal cavity and cause an abscess. Such abscesses may rupture through any of the hollow viscera, the bladder and rectum being the commonest seats of the fistula. But that these abscesses have their origin in the tubes I have several times proved by operation, and in two instances by post-mortem examination.

The question of the conveyance of this disease from men who are apparently cured is one of the utmost importance. I urge upon you the necessity of fully appreciating the importance of this matter. It has been a common observation with me to see a young, healthy, robust girl converted by a year of married life into a pale, suffering, wretched invalid, and this all through the sin of the man who should have done the most for her. Oftentimes, to his credit, it must be said in the majority of cases, it is from his ignorance, and very often from the ignorance of his physician. Many a man is told that it is safe for him to marry, and is given a clean bill of health when he should not have had one.

It is for this very reason, in order that you may be sure of your ground, that I urge upon you to carefully weigh this matter. Nothing is sadder, nor disgusts one more with the sins of humanity, than to see such a case as I have described; and yet they are of almost daily occurrence in my practice. I have in the hospital, now, no less than three such cases, upon whom I have been obliged to perform laparatomy and remove the tubes and ovaries, and in each case the history of the disease is perfectly clear, the women having been perfectly healthy up to the time of their marriage, and the husbands having confessed in each instance to an attack of the disease previous to marriage.

Am I not right, therefore, in insisting that every general practitioner should have the extreme importance of this subject pointed out to him?

There is one matter about which I must say a word, and that is diagnosis. Practitioners, as a rule, have been accustomed to rely upon vaginitis as the distinguishing feature; but, with that sign removed, how are we to distinguish a simple from a specific inflammation? This is often a very puzzling matter, and can only be done in one of two ways: We must either have a straight history of infection, or a probable or possible way in which infection may have occurred; or, failing these, we must resort to bacteriological investigation. There can be no positive proof unless we can find the gonococcus. The inflammation of the vulvo-vaginal gland, as shown by a slight secretion and redness around the mouth of the duct, is held by some to be specific. Others have thought that a urethritis was a constant concomitant; but in the latent form both of these will sometimes fail, as I have myself observed. We may, by induction, conclude that the trouble is gonorrheal if there is a possibility of infection, and if we find the proper lesions in the tubes and ovaries. But this will hardly be considered as a positive proof.

In the male the same thing is true and it may be laid down as a safe rule *no gonococcus*, *no gonorrhea*, *no danger of infection.*

---

THE judge of a certain court in Havre, France, decided recently that an optician who gives a patient advice as to the condition of his eyes and prescribes glasses to remedy defective vision is guilty of the illegal practice of medicine, just as if he had ordered remedies or given medical advice without possessing a diploma recognized by the law.—*Medical Bulletin.*

